# Driving safety: Investigating the cognitive foundations of accident prevention

**DOI:** 10.1016/j.heliyon.2023.e21355

**Published:** 2023-10-21

**Authors:** Jose L. Tapia, Jon Andoni Duñabeitia

**Affiliations:** a Centro de Investigación Nebrija en Cognición (CINC), Universidad Nebrija, Madrid, Spain; b AcqVA Aurora Center, The Arctic University of Norway, Tromsø, Norway

**Keywords:** Driving assessment, Safe driving, Driving performance, Driving simulator, Cognitive abilities

## Abstract

Driving is a crucial aspect of personal independence, and accurate assessment of driving skills is vital for ensuring road safety. This study aimed to identify reliable cognitive predictors of safe driving through a driving simulator experiment. We assessed the driving performance of 66 university students in two distinct simulated driving conditions and evaluated their cognitive skills in decision-making, attention, memory, reasoning, perception, and coordination. Multiple regression analyses were conducted to determine the most reliable cognitive predictor of driving outcome. Results revealed that under favorable driving conditions characterized by good weather and limited interactions with other road users, none of the variables tested in the study were able to predict driving performance. However, in a more challenging scenario with adverse weather conditions and heavier traffic, cognitive assessment scores demonstrated significant predictive power for the rate of traffic infractions committed. Specifically, cognitive skills related to memory and coordination were found to be most predictive. This study underscores the significance of cognitive ability, particularly memory, in ensuring safe driving performance. Incorporating cognitive evaluations in driver licensing and education/training programs can enhance the evaluation of drivers' competence and promote safer driving practices.

## 1 Introduction

Traffic accidents are a pressing concern in developed nations, causing severe harm to both public health and the economy. In 2019, the European Union reported over 22,800 fatalities from road traffic accidents, with 40 % of them happening in urban areas [1]. With each fatality incurring a direct cost of €1.6 million, and serious injuries estimated to cost 13 % of a fatality's value [2], it is crucial to understand the skills and abilities required in the driving process and to identify the factors that most accurately predict accident rates.

In response to these challenges, the emergence of urban infrastructure towards smart cities has represented a promising avenue amidst these worries. These urban zones with innovative technology have been developed to improve the effectiveness and standard of urban services, especially transportation. The incorporation of partially autonomous and completely automated vehicles is a pillar of this strategy. These vehicles are projected to manage metropolitan complexity with less human intervention by utilizing cutting-edge sensors, algorithms, and networking, which promises a major decrease in traffic accidents [3,4]. Accident rates nevertheless persist, demonstrating the significance of comprehending the human component in the equation of driving. In fact, the human factor is at blame for almost 90 % of road accidents [5].

According to contemporary viewpoints, human error is a result of both systemic failure and individual behavior [6], and a comprehensive understanding of these errors necessarily require considering how an individual interacts with their surroundings. Safety-focused cars are ones that support user autonomy and give drivers the least amount of room for accidental error on the road. Even if automation can reduce the risks connected with driving, it will only create new difficulties that can only be foreseen by first comprehending the cognitive processes involved in navigating road traffic systems [7]. This understanding of the broader cognitive landscape sets the stage for a deeper dive into the specific competencies and behaviors that define safe driving.

Driving is a multifaceted activity that necessitates a blend of technical and safety competencies to guarantee a secure and responsible experience on the road. Examining a person's driving techniques and styles is essential to accurately determine their capacity for safe driving [[8], [9], [10]]. In addition to these factors, other critical aspects of safe driving related to the cognitive abilities involved warrant consideration as well. For instance, the concepts of decision-making and risky behavior are closely intertwined and play a pivotal role in driving safety [11]. Each time an individual takes the wheel, they face numerous decisions that can influence not only their own well-being but also the safety of others on the road. Choices such as selecting an appropriate speed, maintaining a safe following distance, and safely navigating intersections are vital for responsible driving [12]. Nevertheless, when drivers engage in risky behavior, such as neglecting traffic regulations, they are more prone to making ill-advised decisions that heighten the likelihood of an accident [13,14].

In a more perceptual and cognitive line, the ability of estimation enables drivers to anticipate the trajectory of their own vehicle and those around them, thus predicting potential collisions. It entails continuous assessment of the surrounding environment and visualization, analysis, and prediction of the paths of other vehicles, pedestrians, or obstacles. Drivers rely on perceptual and subjective variables to approximate speed, time, and distance, which allows them to foresee potential collisions and take suitable actions [15]. Estimation is a complex process involving numerous cognitive elements, such as inhibitory control, working memory, reasoning, planning, and problem-solving [16]. Mastery of estimation skills is essential for gauging distances, foreseeing movements of other vehicles, and determining the appropriate braking time to avert collisions [17].

While each of these factors addresses a specific facet of safe driving, all of them rely on a cognitive foundation as the underlying mechanism. Cognitive abilities, particularly higher-order ones like executive functions, are instrumental in making informed decisions while driving [18]. These skills encompass visual and auditory perception and employ a multitude of cognitive components, including inhibitory control, working memory, reasoning, planning, and problem-solving. Consequently, cognitive abilities have been proposed to be crucial for effectively synthesizing and executing the necessary actions to ensure safe driving practices. Building on this understanding, contemporary research into driving safety increasingly recognizes that the term "human error" is not solely a reflection of individual behavior but is deeply influenced by the broader system in which we operate [19]. This perspective emphasizes that understanding an individual's actions and decisions during driving requires a comprehensive examination of their interaction with the larger driving environment [[20], [21], [22], [23]].

Previous research has examined diverse aspects of cognitive functions and driving performance. Concerning attentional processes, multiple studies have consistently demonstrated a strong correlation between enhanced attentional capabilities and improved driving skills [[24], [25], [26]]. To explore this relationship, researchers have employed various assessment tools, including the Useful Field of View (UFOV), which not only gauges attention but also assesses processing speed and working memory [27]. Moreover, other investigations have employed specific visual attention tests like the Snellen Static Visual Acuity test and the Pelli-Robson Contrast Sensitivity test [28]. Remarkably, these studies have consistently yielded analogous outcomes, further affirming the importance of attention in predicting the on-road driving performance [29]. Likewise, research centered on perceptual abilities has consistently demonstrated their significance as reliable predictors of driving performance. Specifically, assessments related to 3D motion perception have proven to be robust indicators of braking response time, offering valuable insights into a driver's ability to react swiftly to changes in the road environment [30]. Additionally, when it comes to hazard perception, the aptitude for velocity discrimination in moving objects emerges as a particularly reliable predictor, shedding light on a driver's capacity to identify potential dangers on the road ahead [31]. In the context of memory, there exists a clear connection with driving proficiency, especially when engaged in multitasking situations, as evidenced by a preliminary investigation using functional near-infrared spectroscopy (fNIRS) and eye tracking [32]. Moreover, the capacity of working memory has been pinpointed as a strong predictor for a variety of navigation-related tasks and notably, the occurrence of safety-related errors during driving [33]. Furthermore, some researchers suggest that individuals with greater memory skills may display a reduced inclination for distracted driving, highlighting the critical function of memory in maintaining focused driving [34].

The various aspects of cognition that have been revealed by research so far have helped to clarify how they each affect driving performance. It is undeniable that a driver's competence and safety on the road can be affected by attentional processes, perceptual abilities, and memory. Nevertheless, despite these discoveries, there is still a sizable knowledge gap regarding the complex interactions and overlap among these cognitive components. The subtle interactions between attention, perception, and memory while driving remain largely unexplored territory. Therefore, further research is necessary to fully understand how these cognitive domains interact and collectively influence driving behavior given the complexity inherent in these relationships.

### 1.1 The current proposal

The proposed study seeks to thoroughly explore the intricate interplay among cognitive variables to identify the most accurate predictors of safety driving. To achieve this goal, the study will assess factors such as decision-making, attention, perception, memory, reasoning, and coordination using widely recognized digital instruments and a driving simulator. By incorporating diverse testing conditions and scenarios, the study aims to better understand the roles these variables play in predicting traffic infractions and ultimately improve road safety. The central hypothesis of the study is that cognitive assessment will be crucial in promoting driving safety, and the findings will support the development of targeted interventions and strategies aimed at reducing the occurrence of traffic accidents and their adverse economic, health, and social consequences.

## 2 Material and methods

### 2.1 Participants

A total of 66 university students aged between 18 and 35 years (M = 22.21; SD = 3.13; 41 % of females) participated in the data collection process. All participants were residents of the metropolitan area of Madrid (Spain) and had a valid B-category driving license for a mean period of 2.5 years (SD = 1.55), and with an annual mileage of 6371 km (SD = 6364). Table 1 offers a breakdown of these statistics by gender. Three of the participants also had a motorcycle license. The experimental procedure was approved by the Ethics Board of Universidad Nebrija (UNNE-2021-007) and conformed to the Declaration of Helsinki.

**Table 1:** Mean and standard deviation of age, license duration, and annual mileage by gender.

Gender	Age (in years)		License duration (in years)		Annual milage (in kilometers)	
	Mean	SD	Mean	SD	Mean	SD
Male	23.23	3.41	2.95	1.57	6961.54	6639.58
Female	20.74	1.89	1.93	1.33	5518.52	5962.19

### 2.2 Assessment instruments

In shaping our research design, we strategically incorporated three assessment tools to comprehensively understand the nexus between cognitive functions and driving behavior. We initiated with a driving simulator, meticulously designed to mirror real-world driving scenarios within a controlled setting [[35], [36], [37]]. This not only ensured the authenticity of participants' responses but also eliminated the inherent risks of on-road evaluations. Complementing this, we introduced a decision-making task tailored to gauge participants' decision-making processes in high-risk situations—a skill directly translatable to safe driving practices [38,39]. Concluding our assessment suite was a robust cognitive evaluation that spanned multiple cognitive domains. This was essential in crafting a detailed cognitive profile for each participant, laying the groundwork for discerning correlations between specific cognitive abilities and their driving performance [40,41].

**Driving simulator:** As a driving simulator the Simumak® Simescar lite version open cockpit was used. The simulator environment was installed on Windows 10 (64 bits) HP EliteDesk 800 G5 TWR i5 computer, 16 Gb RAM, NVIDIA GeForce GTX 1660 Ti video card and displayed on three 27” IPS Full HD HP monitors, providing a horizontal physical 180° field of view. The simulation environment consisted of a 4-km guided urban drive, with ongoing traffic, cyclists and pedestrians. Participants had to follow the directions of the navigation system, starting from a parking lot, driving 2.5 km in an urban area, 1 km of interurban route and 0.5 km in a residential area, where they had to park and finish the exercise. The speed limits were 40 km/h in urban areas and 60 km/h on interurban roads. In accordance with the scenario previously described, two distinct driving conditions were formulated to simulate contrasting realistic situations. The simulated driving condition 1 (SDC1) was configured to replicate a sunlit day with low traffic volume, limited pedestrian presence, and predictable driving conduct of other vehicles. Conversely, the simulated driving condition 2 (SDC2) was devised to replicate a scenario featuring moderate rainfall, decreased visibility, intensified traffic, and greater presence of pedestrians and cyclists, alongside the reduced predictability of other vehicles' behavior (i.e., unexpected braking and lane changes without the use of turn signals). Although the behavior of other vehicles and pedestrians was characterized by random and fluctuating behavior in both conditions, specific events that could lead to hazardous circumstances were integrated into each condition. For scoring, we considered the occurrence of traffic infractions including failure to use turning signals, failure to give priority to other vehicles, bicycles, or pedestrians, failure to maintain a safe distance from cyclists, exceeding the maximum speed limit, driving too fast in adverse weather conditions, swerving out of the lane, disregarding red lights and stop signs, driving in the wrong direction, and collisions.

**Decision making:** To assess risky decision making in an uncertain environment, we used a computerized version of the Iowa Gambling Task (IGT) [42]. The task required participants to select cards from four decks of cards (A, B, C, D) with each card having a gain or loss value. Participants were unaware of the exact amount of money that could be gained or lost by choosing each card. Two of the decks (C and D) had positive expected values, meaning gains were greater than losses in the long run, while the other two (A and B) had negative expected values, meaning losses were greater than gains in the long run. The task included a total of 100 trials, and the score was calculated by summing the total number of times participants selected decks A and B. No time constraints were applied.

**Cognitive assessment:** The Spanish version of the Driving Cognitive Assessment (DAB)® (CogniFit Inc., San Francisco, CA, USA) was used to assess the cognitive function of the participants and to obtain a complete cognitive profile. The assessment battery has a total of 12 computer tasks measuring 5 main cognitive domains (attention, memory, reasoning, perception, and coordination) and 22 cognitive skills. The cognitive domain of attention encompasses the cognitive skills of inhibition, divided attention, focus attention, and updating. Memory includes the skills of working memory, visual and auditory short-term memory, visual memory, contextualized memory, and naming skills. Reasoning is composed of processing speed, planning, and shifting. Perception consists of visual scanning, visual, spatial, and auditory perception, recognition, and estimation. Coordination integrates eye-hand coordination and response time. A gender- and age-adjusted z-score for each cognitive domain, and overall cognitive performance was used.

### 2.3 Procedure

Participants were recruited through internal university communications and social media. Those who expressed interest in the study were invited to the laboratory facilities, where they received detailed information about the study and provided informed consent. The experimental session was conducted individually in a quiet room to minimize distractions. The research team monitored the procedure from an adjacent room. Task order was randomized, and a 5-min break was provided between tasks. The IGT was designed on Gorilla Experiment Builder (www.gorilla.sc) [43]. Both the IGT and the DAB were displayed on a Lenovo Yoga 730 laptop equipped with an Intel Core i5-8250U processor, 8 GB of RAM, and a UHD 620 video card. Prior to the driving simulator evaluation, participants received training on the simulator hardware and completed a 3-min unrestricted driving session to acclimate to the mechanical characteristics and environment of the simulator.

## 3 Results

### 3.1 Data processing

The collected data were processed using Rstudio [44] and analyzed with JASP [45]. Initially, a series of correlation analyses were conducted to determine the possible relationships between the number of years since the obtention of the driving license and the millage per year on traffic infractions committed. Following these correlation analyses, paired-sample t-tests were performed to assess the differences in the number of driving infractions between the two driving conditions. Subsequently, a series of multiple regression analyses were carried out to investigate the predictive power of the assessment instruments and explore any potential gender-based differences in driving infractions. The subsequent section provides a comprehensive overview of the statistical analyses conducted and their corresponding results.

### 3.2 Correlation analyses

To understand the factors influencing driving infractions, we examined the relationship between driving experience (measured as the number of years in possession of the driving license, and as the number of kilometers driven per year), and the number of infractions in each simulated driving scenario and overall. There was a very weak and non-significant correlation between the number of years in possession of the driving license and the number of infractions in the two separated scenarios (SDC1: r = −0.029, p = .817; SDC2: r = 0.002, p = .987), or the overall (summed) number of infractions (r = −0.012, p = .925), indicating no reliable trend for those with longer license durations to have fewer infractions. In contrast, a close-to-significant correlation was observed between the number of kilometers driven per year and the number of infractions in the more demanding scenario and overall (SDC1: r = −0.178, p = .153; SDC2: r = −0.226, p = .068; Overall: r = −0.221, p = .075). This suggests that participants driving more kilometers annually are more likely to have a lower number of infractions, plausibly due to their increased driving experience.

### 3.3 Simulated driving condition comparison

**Fig. 1:**
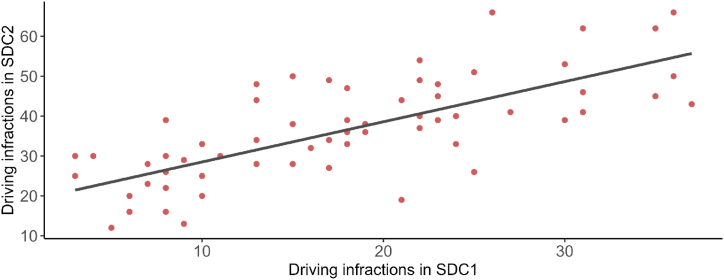
Relationship between driving infractions in the two simulated driving conditions. Note. The x-axis displays the number of infractions committed in Simulated Driving Condition 1 (SDC1), while the y-axis shows the number of infractions committed in Simulated Driving Condition 2 (SDC2). Each point in the plot represents a participant's driving behavior under both conditions. The line indicates the linear regression model fitted to the data, which reflects the positive correlation between infractions committed in the two driving scenarios.

A paired-sample *t*-test was conducted to compare the number of driving infractions between the two experimental driving conditions, SDC1 and SDC2. As expected, the mean number of infractions was significantly lower in SDC1 (M = 17.56, SD = 9.17) than in SDC2 (M = 36.12, SD = 12.60); t (65) = 17.59, p < .001, d = −2.17. Appendix 1 provides a comprehensive frequency list of all traffic infractions by gender and simulated driving condition. Furthermore, a correlation analysis revealed a strong and significant positive correlation between SDC1 and SDC2 (r = 0.733, p < .001). This indicates that participants who committed more infractions in one condition also tended to do so in the other, showing the consistency of individual driving performance across both simulated conditions (Fig. 1).

### 3.4 Regression analyses of assessment instruments as predictors of driving outcomes

To determine the relationship between cognitive variables and driving infractions, we conducted regression analyses that included both assessment instruments and gender as predictor variables for driving infractions in the two simulated driving conditions. The factor Gender was included to account for potential gender-based differences in driving behavior, as suggested by previous research [46].

For the first simulated driving condition (SDC1), the dependent variable was the number of driving infractions and the predictors were the assessment instruments (namely, IGT and DAB) and gender, with the interactions between the instruments and gender being also included (see Table 2). The regression model was not statistically significant (R2 = 0.10, F (5, 65) = 1.34, p = .261), and none of the predictors had a significant effect on predicting driving infractions.

**Table 2:** Regression analyses results for simulated driving condition 1.

Predictors					95 % CI	
	β	SE	t	p	Lower	Upper
DAB	−8.98	4.71	1.91	.061	−18.39	0.44
IGT	0.01	0.08	0.10	.925	−0.14	0.16
gender	−1.18	7.08	0.17	.868	−15.34	12.97
DAB * gender	7.20	7.99	0.90	.372	−8.80	23.19
IGT * gender	0.07	0.13	0.27	.787	−0.23	0.30

A similar approach was followed for the data obtained in the second simulated driving condition (SDC2; see Table 3). The regression model demonstrated marginal statistical significance (R2 = 0.16, F (5, 65) = 2.32, p = .054). A significant effect of DAB in predicting participants' driving outcomes was found (β = −16.99, 95 % CI [−29.48, −4.50], t (60) = 2.72, p = .009), with participants with higher cognitive abilities showing fewer driving infractions. Furthermore, although the effect of gender was not significant, there was a significant interaction between gender and DAB, β = −21.5, 95 % CI [0.37, 42.80], t (50) = 2.04, p = .046. This interaction suggests that the relationship between cognitive abilities (DAB) and driving infractions differs between men and women. Specifically, men with higher cognitive profiles tended to commit fewer infractions, while the cognitive profile of women did not predict the number of driving infractions. No other interaction was significant.

**Table 3:** Regression analyses results for simulated driving condition 2.

Predictors					95 % CI	
	β	SE	t	p	Lower	Upper
DAB	−16.99	6.24	2.72	.009**	−29.48	−4.50
IGT	−0.07	0.10	0.70	.490	−0.27	0.13
gender	−4.84	9.39	0.52	.608	−23.62	13.94
DAB * gender	21.59	10.61	2.04	.046*	0.37	42.80
IGT * gender	−0.01	0.18	0.04	.971	−0.36	0.34

Note. *p < .05; **p < .01.

**Fig. 2:**
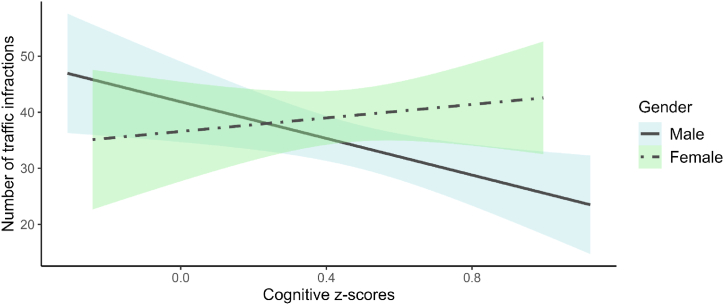
Relationship between cognitive ability and traffic infractions by gender. Note. The figure shows the relationship between cognitive ability, measured by standardized z-scores, and the number of traffic infractions committed in the simulated driving condition 2, stratified by gender. The graph reveals a negative association between cognitive ability and traffic infractions for males, meaning that as cognitive ability increases, the number of traffic infractions decreases. In contrast, for females, this relationship did not exist.

As a following step we conducted a second simplified model that included SDC2 driving infractions as the dependent variable and only the DAB scores, gender, and their interaction as predictive variables. This model accounted for a statistically significant amount of variance (R2 = 0.15, F (3, 65) = 3.68, p = .017). DAB scores had a statistically significant and negative effect (β = −16.34, 95 % CI [−28.55, −4.12], t (62) = −2.67, p = .010), indicating that higher cognitive performance was associated with fewer traffic infractions. The interaction between DAB scores and gender was statistically significant (β = 22.36, 95 % CI [2.15, 42.57], t (62) = 2.21, p = .031), where higher cognitive ability scores were associated with fewer driving infractions for men, whereas this was not the case for women (see Fig. 2).

To better understand the influence of gender on the results, we conducted further analysis by fitting distinct linear models for male and female participants. The results of the analysis revealed that the linear model used to predict SDC2 driving infractions with DAB scores for males accounted for a statistically significant and moderate proportion of variance (R2 = 0.16, F (1, 37) = 7.11, p = .011). The effect of DAB was found to be statistically significant (β = −16.34, 95 % CI [−28.75, −3.92], t (37) = −2.67, p = .011. Nevertheless, the model including SDC2 driving infractions and DAB scores for females explained a negligible proportion of variance (R2 = 0.02, F (1, 25) = 0.56, p = .460). The effect of DAB scores was statistically non-significant (β = 6.02, 95 % CI [−10.50, 22.55], t (25) = 0.75, p = .460.

### 3.5 Regression analyses of cognitive assessment as predictors of driving outcomes

In order to explore which cognitive domain explained most of the variance among diving infractions a main general regression model was constructed. The model included SDC2 driving infraction scores as dependent variable and the five DAB cognitive domains as predictor variables (i.e., attention, memory, reasoning, perception, and coordination). Gender was included as predictor and control variable for between-gender differences; thus, the interaction between gender and the domains were also included in the regression model.

**Fig. 3:**
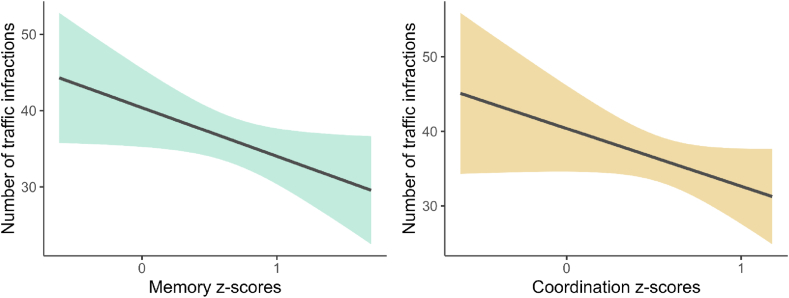
Relationship between memory and coordination subscales and traffic infractions. Note. The graphs illustrate the mean scores and corresponding confidence intervals for two cognitive domains, memory (left graph) and coordination (right graph), among study participants. The observed trend shows a decrease in the number of traffic infractions as cognitive ability increases in both domains.

The model explained a statistically significant and large proportion of variance (R2 = 0.29, F (11, 65) = 2.02, p = .044). The effect of memory and coordination subscales were statistically significant and negative; β = −13.98, 95 % CI (−24.19, −3.77), t (54) = −2.74, p = .008, and β = −22.36, 95 % CI (−41.57, −3.15), t (54) = −2.33, p = .023; respectively (see Fig. 3). Therefore, higher scores on memory and coordination reflected lower driving infractions. No other domain showed a statistically significant effect. Gender showed no statistically significant effect β = −11.67, 95 % CI (−26.65, 3.30), t (62) = −1.56, p = .124. Finally, no significant interactions were found between gender and memory, nor between gender and coordination.

## 4 Discussion

The present study investigated the impact of decision-making and cognition on safe driving practices, by administering well-validated assessment tools to a sample of novice drivers in two distinct driving conditions. The findings suggest that while decision-making may not be a significant predictor of driving outcomes as previously stated [7], cognitive assessment scores can predict driving infractions in challenging driving conditions, supporting the notion that cognitive abilities are important determinants of driving performance. Gender was not found to have a significant main effect on driving infractions, but an interaction between cognitive scores and gender was observed, highlighting potential gender-based differences in the effect of cognition on driving performance. The amount of time a person has held their driving license did not correlate with driving performance. However, the approximate mileage driven per year did, suggesting that experienced drivers – namely those who cover more kilometers per year - show a better driving performance.

Delving into decision-making, in line with our results, previous studies also found non-significant predictability of the Iowa Gambling Task on driving [47], suggesting that a person's propensity to take risky situations involving finances may differ from those taken while driving, where physical integrity may be compromised. Nevertheless, the high predictability power found for cognitive abilities underscores the potential usefulness of cognitive assessments in identifying individuals who are more susceptible to driving infractions and reinforces the growing body of literature that indicates the importance of cognitive abilities in driving performance [18,48]. While gender was not found to be a significant factor in driving behavior our results suggest that the relationship between cognitive ability and driving infractions may differ between men and women. Specifically, in the case of females, cognitive abilities do not seem to modulate the number of traffic infractions they made, whereas in the case of males cognitive abilities played a relevant role on the driving outcome (i.e., higher cognitive abilities predicted lower number of infractions). These findings indicate that there might be factors beyond cognitive abilities that may contribute to driving behavior such as personality traits, emotion or risk perception. For instance, gender differences have been identified in teenage drivers' self-perceptions of safe driving behaviors and self-reported risk behaviors while driving [49], as well as in risk-taking judgments [50]. Additionally, driving styles are known to vary between genders and age groups and correlate with personality traits [51,52].

Beyond theoretical knowledge and practical driving skills, real-life driving situations require cognitive functions such as monitoring various stimuli, analyzing environmental information, and adapting to unexpected situations. Therefore, it is not surprising to have found a high predictive power of cognitive skills in driving outcome. As per the specific cognitive abilities our results are consistent with the literature's assertion that memory and coordination may be determining factors in driving [[53], [54], [55]]. Specifically, the memory cognitive domain emerged as the most relevant predictor of the number of infractions committed while driving on a simulator. This domain is closely related to executive functions and includes cognitive skills that may be essential for driving, such as short-term memory, contextual memory, and working memory [56,57]. Memory thus encompasses a wide range of skills such as extracting and retaining surrounding information, contrasting that information with prior knowledge and experiences, analyzing the current situation, and processing and manipulating information in real-time (i.e., adjusting speed and distance from other vehicles). Moreover, our results also show coordination to significantly predict driving outcome, as this skill is especially important in emergency situations that require quick and precise action [58]. For instance, performing an evasion maneuver to avoid an obstacle on the road requires excellent coordination between visual perception, cognition, and motor skills.

Despite the promising results, several limitations should be considered. First, our sample included only university students, lacking experienced drivers. Second, we only took into account the number of traffic infractions while ignoring other factors that could be determined from the vehicle's telemetry, such as the driver's skill with the accelerator and brake pedals and their smoothness when handling the steering wheel. Furthermore, it is important to note that cognitive abilities progressively decrease with age [15,[59], [60], [61]]. As a result, to extend these findings to different population groups, further studies are necessary. Moreover, even though driving simulators are increasingly being used in training novice drivers and assessing driving performance [62], there is no standardized hardware or software that enables comparison of the findings across studies. Hence, developing a platform with validated specifications and scenarios would benefit the generalizability of results. Additionally, it is worth nothing that our experiment was conducted entirely in simulation, so the reliability of our results would be enhanced by an evaluation off-road.

Although we emphasize the association of the memory domain with executive functions, it is noteworthy that the DAB's attention domain encompasses components like inhibition and updating. These components are traditionally attributed to executive functions [63]. Yet, despite this alignment, the attention domain did not significantly influence our regression analysis. This discrepancy warrants a deeper exploration into how the DAB measures memory and attention domains. The interplay between these domains is evident. For instance, tasks like working memory, categorized under DAB's memory domain, inherently demand attentional resources. This suggests that while the DAB differentiates between memory and attention, their real-world application, especially in intricate tasks like driving, might blur these distinctions. It is possible that our study's tasks required both attention and memory skills, but the memory domain had a more pronounced effect on driving performance. The specific driving scenarios we employed might have emphasized memory processes, such as recalling past traffic scenarios or anticipating other vehicles' trajectories, over pure attentional tasks. This could account for why memory emerged as a significant predictor in our study, overshadowing other cognitive components within the attention domain.

Reflecting on our findings, we acknowledge the multifaceted nature of driving. While our research emphasizes the roles of memory and coordination, driving is influenced by a broader range of factors encompassing cognitive, behavioral, psychological, and socio-cultural dimensions. Our study offers insights into specific cognitive domains but does not address all the nuances shaping driving behaviors. Individual driving patterns, shaped by diverse experiences and cultural backgrounds, have profound implications for road safety [64]. Socio-cultural norms, deeply embedded within societal structures, can influence driving behaviors comparably to cognitive or physiological factors [65,66]. Furthermore, on-road decisions may be manifestations of deeper personality structures, ranging from inherent risk-taking tendencies to more contemplative approaches [[67], [68], [69]]. Behavioral tendencies, influenced by societal expectations and peer behaviors, are also significant [70]. Our study, therefore, emphasizes certain facets while not fully exploring the comprehensive, complex domain of driving behaviors. This complexity, shaped by personality, behavior, and societal norms, necessitates a broader research perspective.

In light of these results, there are indications to propose a renewal of current training programs and incorporate cognitive aspects into them. Thus, driver training modules should incorporate simulation scenarios that intensively test and develop cognitive capacities, especially those related to memory and coordination. For example, exercises could be designed where learners are required to quickly recall traffic rules and regulations in dynamically changing environments, akin to real-life driving situations. These exercises might also emphasize predicting potential actions of other drivers or pedestrians based on previously observed patterns. Likewise, scenarios could involve processing multiple pieces of information simultaneously, such as monitoring changing traffic lights, reacting to sudden moves from other vehicles, and adhering to signaled directions. Moreover, given the significance of coordination in our findings, training should also incorporate tasks that demand the synchronization of visual perception, cognition, and motor skills. Learners could be exposed to scenarios that test their reflexes and coordination, like evasive maneuvers to avoid sudden obstacles or effectively navigating through tight spaces, mimicking real-life emergency situations. By honing these specific cognitive domains through targeted training, new drivers will not only acquire the essential theoretical-motor skills but will also develop crucial cognitive agility for safe and effective driving. The dual focus on memory and coordination underscores a more comprehensive approach to driver training, emphasizing the multifaceted nature of driving that goes beyond mere physical control of the vehicle. Incorporating these elements into training will foster a generation of drivers who are better prepared mentally and physically for the challenges of the road.

These findings have important implications for identifying individuals who may be at risk of unsafe driving behavior and developing effective training programs to promote safe driving. In particular, by identifying cognitive deficits early on, targeted interventions can be developed to address these areas before granting a driving license. To further improve traffic safety, ongoing research is focused on developing assessment methods for continuous evaluation of driving skills. By incorporating cognitive testing and training, it may be possible to accelerate the driving learning process and reduce the risk of accidents. Moreover, regular re-evaluation throughout a driver's lifetime might identify cognitive decline or other risks before they become a threat on the road. Including cognitive assessments in the driver licensing process could also reduce traffic infractions and improve overall road safety. Such assessments could become a standard component of driver's education, ensuring that drivers are not only knowledgeable about road rules but are also cognitively equipped to make safe driving decisions.

Building on this, future studies should investigate the role of cognitive variables for other age groups and determine whether the cognitive skills involved in driving are constant for novice, experienced, or professional drivers. For example, older drivers might have declining cognitive functions but more experience, which could impact their driving differently compared to novice drivers. Further research could also explore the relationship between other simulation parameters and cognitive function. These could include aspects like reaction time in high-pressure simulations or multitasking abilities in complex driving scenarios. Additionally, larger sample sizes in driving simulator studies will improve the external validity of the findings and lead to more generalizable and reliable results. It may also be worthwhile to investigate cross-cultural differences in cognitive abilities and driving behavior, offering insights into how varying societal norms and driving environments interact with cognition. Overall, these future investigations will enhance our understanding of the role of cognitive abilities in driving and aid in the development of interventions to promote safer driving behavior.

## 5 Conclusion

In conclusion, this study highlights the significant impact of cognitive abilities on safe driving practices. While decision-making was not a strong predictor, cognitive assessment scores demonstrated predictive power for driving infractions, particularly in challenging driving conditions. Gender differences were observed in the relationship between cognitive abilities and driving performance. Memory and coordination emerged as crucial cognitive domains, emphasizing their importance in real-life-like driving situations. These findings have implications for identifying at-risk drivers and improving road safety through cognitive testing and training. Future studies should further explore cognitive variables across different driver groups and investigate the relationship between driving parameters and cognitive function to enhance our understanding of the role of cognitive abilities in driving and develop effective interventions.

## Funding

The research project is founded by the grant ISERI from the “Ayudas Fundación 10.13039/501100005142BBVA a Proyectos de Investigación Científica 2021” and grants FPU19/02239; 2020–2024 and PID2021-126884NB-I00 by the MCIN/10.13039/501100011033AEI/10.13039/501100011033.

## Data availability

The data supporting the findings of this study have not been deposited into a publicly available repository in accordance with the agreement established with the individuals who participated and signed the consent form. Nevertheless, anonymized aggregated data can be provided upon a reasonable request to the corresponding author.

## CRediT authorship contribution statement

**Jose L. Tapia:** Conceptualization, Data curation, Formal analysis, Investigation, Methodology, Project administration, Software, Validation, Visualization, Writing – original draft, Writing – review & editing. **Jon Andoni Duñabeitia:** Conceptualization, Data curation, Formal analysis, Funding acquisition, Investigation, Methodology, Project administration, Resources, Software, Supervision, Validation, Visualization, Writing – review & editing.

## Declaration of competing interest

The authors declare that they have no known competing financial interests or personal relationships that could have appeared to influence the work reported in this paper.

## Figures and Tables

**Table:** 

Gender	Simulated driving condition	Failure to use turning signals	Failure to give priority to other vehicles	Failure to give priority to pedestrians	Failure to give priority to bicycles	Failure to maintain a safe distance from cyclists	Disregarding red lights and stop signs	Exceeding maximum speed limit	Speeding in adverse weather conditions	Swerving out of the lane	Driving in the wrong direction	Collision with vehicle	Collision with pedestrian	Collision with bicycle
Male	SDC1	59	2	1	32	7	41	417	0	64	2	2	0	0
Male	SDC2	67	52	7	61	50	50	357	601	46	14	13	0	4
Female	SDC1	36	11	1	30	2	31	279	0	131	6	5	0	0
Female	SDC2	31	38	3	61	52	31	289	441	84	16	13	0	3

Note: This table presents the frequency of traffic violations, categorized by gender and simulated driving condition. The values represent the total number of observed infractions for each combination of gender (Male/Female) and simulated driving condition (SDC1/SDC2).
